# Characteristics and Properties of Acid- and Pepsin-Solubilized Collagens from the Tail Tendon of Skipjack Tuna (*Katsuwonus pelamis*)

**DOI:** 10.3390/polym14235329

**Published:** 2022-12-06

**Authors:** Sagun Chanmangkang, Sutee Wangtueai, Nantipa Pansawat, Pramvadee Tepwong, Atikorn Panya, Jirawan Maneerote

**Affiliations:** 1 College of Maritime Studies and Management, Chiang Mai University, Samut Sakhon 74000, Thailand; 2 Department of Fishery Products, Faculty of Fisheries, Kasetsart University, Bangkok 10900, Thailand; 3 Food Biotechnology Research Unit, National Center for Genetic Engineering and Biotechnology, Pathum Thani 12120, Thailand

**Keywords:** collagen, tuna-tail tendon, *Katsuwonus pelamis*, collagen properties

## Abstract

The tail tendons of skipjack tuna (*Katsuwonus pelamis*), a by-product from the meat-separation process in canned-tuna production, was used as an alternative source of collagen extraction. The acid-solubilized collagens using vinegar (VTC) and acetic-acid (ATC) extraction and pepsin-solubilized collagen (APTC) were extracted from tuna-tail tendon. The physiochemical properties and characteristics of those collagens were investigated. The obtained yield of VTC, ATC, and APTC were 7.88 ± 0.41, 8.67 ± 0.35, and 12.04 ± 0.07%, respectively. The determination of protein-collagen solubility, the effect of pH and NaCl on collagen solubility, Fourier-transform infrared spectroscopy (FTIR) spectrum, and microstructure of the collagen-fibril surface using a scanning electron microscope (SEM) were done. The protein solubility of VTC, ATC, and APTC were 0.44 ± 0.03, 0.52 ± 0.07, and 0.67 ± 0.12 mg protein/mg collagen. The solubility of collagen decreased with increasing of NaCl content. These three collagens were good solubility at low pH with the highest solubility at pH 5. The FTIR spectrum showed absorbance of Amide A, Amide B, Amide I, Amide II, and Amide III groups as 3286–3293 cm^−1^, 2853–2922 cm^−1^, 1634–1646 cm^−1^, 1543–1544 cm^−1^, and 1236–1237 cm^−1^, respectively. The SEM analysis indicated a microstructure of collagen surface as folding of fibril with small pore.

## 1. Introduction

Tuna-processing industry is the most important of fishery-product processing in the world. About 75% of tuna world capture is supplied for canning productions that have a skipjack (*Katsuwonus pelamis*) as one of major commercial species [1]. Over 60% by-products are left from canned-tuna processing, such as head, bone, gill, dark meat, blood, viscera, skin, tail tendon, and others [2]. Several processes have been used to utilize these by-products with high value creation as bioactive ingredients and/or nutritional supplements for health benefits [3]. Tuna-tail tendons are generally a by-product that is obtained from the meat-separation process in canned-tuna processing. The main composition of tuna-tail tend has been identified as containing about 82% of type I collagen [4]. It could be used as a potential alternative raw material for marine-collagen extraction.

Collagen is the major basic protein within the connective tissue of vertebrates and constitutes approximately 30% of the whole animal protein. Collagen is known as a stoma or fibrous protein, contributing to specific physiological function of tissues in skins, tendons, bones, and cartilages [5]. Collagen comprises different types of amino acids and are arranged in a specific manner with various bonds making stronger and more stable structure [6]. Currently, approximal 28 different collagen types have been identified and characterized. They each differs greatly in amino-acid sequence, structure, and function, which are mostly a type I collagen [7,8]. These are distributed in different tissues, such as type-I in dermis, bones, cornea, ligaments, and tendons [9]; type-II is in the vitreous body and cartilage; type-III is in reticular fibers of lungs, vessels wall, spleen, and liver; type-IV is in basement membranes and type-V is co-distributed with type-I i.e., cornea. Type-I collagen is the most commonly used as a biomedical material for wounds dressing, tissue- engineering construct, drug-delivery system, and cosmetics because it causes less allergy and has the ability to adhere well to cells [10,11]. Regarding a wide application of collagen, there is an increasing demand with the predicted global collagens market size in 2025 to reach ~6.63 billion USD [12]. In commercial production, collagen is normally obtained from mammal skins and bones. However, alternative collagenous materials have become more interesting to obtain the collagen, such as fish skin, bone, scale, and other by-product from the fish-processing industry. These by-products are used to extract collagen by using two important processes, including raw-material preparation and extraction [13]. Generally, the important variables for collagen extraction are yield and several properties, which are affected by various factors, such as concentration of acid or enzyme, extraction time, temperature, ratio of acid solutions and raw materials, collagenous-material type, and others [14]. 

According to previous research, the various studies on the characterization and functional of extracted collagen from a variety of marine source by-products have been reported. The collagen from tuna by-products has been isolated and characterized, such as tuna skins [15,16,17], spines and skulls [18], bones [19], and swim bladders [20]. However, to date, the report on the extraction and characterization of collagen from the tail tendon of tuna is much less explored. Thus, the objective of this study was to extract and characterize the acid-solubilized collagens using vinegar (VTC), acetic-acid (ATC) extraction, and pepsin-solubilized collagen (APTC) from tail tendons of skipjack tuna (*Katsuwonus pelamis*).

## 2. Materials and Methods

### 2.1. Raw Material and Preparation

The tail tendons of skipjack tuna (*Katsuwonus pelamis*) were obtained from the process of meat separation from canned-tuna production (Global Pet Care Business Unit, Thai Union Group PCL., Samut Sakhon, Thailand). After collection, tuna-tail tendons were packed in a plastic bag, frozen, and transported to the laboratory of College of Maritime Studies and Management, Chiang Mai University, Samut Sakhon Province within 20 min. During transportation, frozen tuna-tail tendons were kept in insulated box with ice (ratio of ice: raw material 2:1). Upon arrival, the sample was stored at −18 to −20 °C until further use in the experiment (not exceeding 4 months). Before extraction, the tendons were cut into small piece using a knife by hand and chopped using silent cutter (Daily Collection HR7627 Blender, Philips, Bangkok, Thailand). To eliminate non-collagenous from the raw material, the chopped tail tendons were soaked in 100 mM NaCl (pH 7.5) for 12 h, washed in distilled water, and subjected to collagen extraction.

### 2.2. Chemicals and Enzyme

Pepsin (activity ≥ 3200 U/mg protein), β-mercaptoethanol (β-ME), 2,2-diphenyi-l-picrylhydrazyl radical (DPPH), 2,2′-azinobis-(3-ethylbenzthiazoline-6-sulfonic acid) (ABTS), 2,4,6-tripyridyl-s-triazine (TPTZ), ferric chloride hexahydrate (FeCl_3_·6H_2_O), N-tert-butyldimethylsilyl-N-methyltrifluoroacetamide (MTBSTFA), and 1% tert-butyldimethylchlorosilane (TBDMSCl) were purchased from the Sigma-Aldrich Co. (St. Louis, MO, USA). High molecular weight (MW) markers were obtained from GE Healthcare UK (Aylesbury, UK). Sodium dodecyl sulphate (SDS) and Coomassie Blue R-250 were procured from Bio-Rad Laboratories (Hercules, CA, USA). Sodium chloride and acetic acid were purchased from Merck KGaA (Darmstadt, Germany). Other chemicals and reagents used were analytical grade.

### 2.3. Tuna Tail Tendon Collagen Extraction

#### 2.3.1. Extraction of Acid-Solubilized Collagen Using Vinegar Extraction

The acid-solubilized collagen using vinegar extraction was done according to the modification method of Tamilmozhi et al. [21]. The pretreated tuna-tail tendons (from 2.1) were soaked in 5% vinegar (Kewpie (Thailand) Co., Ltd., Bangkok, Thailand) with the ratio of tendon: 5% vinegar 1:20 (*w*/*v*) at 10 ± 1 °C using shaking incubator (LSI-3016R, Daihan Labtech Co., Ltd., Gyeonggi-do, Republic of Korea) with 200 rpm for 72 h. The extracted solution was filtrated using a double layer of cheesecloth and kept at 4 °C. The pellet was re-extracted with the same manner. The extracted solutions were mixed and precipitated using NaCl adding with a final concentration of 2.6 M for collagen obtaining. The resulting precipitate was obtained by centrifugation at 10,000× *g* for 30 min at 4 °C. Thereafter, the pellet was resuspended in a minimum volume of 5% vinegar (1:10, *w*/*v*), followed by dialysis in 50 volumes of distilled water for 24 h using the 100 kDa dialysis membrane (Spectra/Por, Thermo Fisher Scientific Inc., Gothenburg, Sweden). The resulting dialysate was lyophilized using a freeze-dryer (GFD-3H, Grisrianthong, Samut Sakhon, Thailand) to obtain acid-solubilized collagen using vinegar extraction (VTC) and kept at −18 to −20 °C until further use.

#### 2.3.2. Extraction of Acid-Solubilized Collagen Using Acetic Acid Extraction

The acid-solubilized collagen using acetic-acid extraction was done according the method of Tamilmozhi et al. [21] with a slight modification. The pretreated tuna tendons were soaked in 0.5 M acetic acid (1:10, *w*/*v*) and stirred using shaking incubator (LSI-3016R) with 200 rpm for 48 h at 10 ± 1 °C. The extracted solution was filtrated using a double layer of cheesecloth and kept at 4 °C. The pellet was re-extracted with the same manner. The extracted solution was precipitated using NaCl with the same manner of VTC (Section 2.3.1). The precipitate was dissolved in 0.5 M acetic acid and then dialyzed against the distilled water for 24 h using the 100 kDa dialysis membrane (Spectra/Por, Thermo Fisher Scientific Inc.). The resulting dialysate was freeze-dried and considered the acid-solubilized collagen using acetic-acid extraction (ATC). The ATC was stored at −18 to −20 °C until further use. 

#### 2.3.3. Extraction of Pepsin-Solubilized Collagen

The pepsin-solubilized collagen was carried out using the modified method of Tamilmozhi et al. [21]. The pretreated tuna tendon was stirred in a mix solution of 0.5 M acetic acid and 1% (*w*/*w* protein) pepsin using a tendon/solution ratio of 1:10 (*w*/*v*) for 48 h at 10 ± 1 °C in a shaking incubator (LSI-3016R) with 200 rpm. After extraction, the supernatant was obtained in the same previous mention for VTC preparation. The precipitation, dialysis, and freeze-drying were carried out in the same manner with VTC. Then, pepsin-solubilized collagen (APTC) was obtained and kept at −18 to −20 °C until further use. 

### 2.4. Analyzes

#### 2.4.1. Proximate Compositions

Moisture, protein, lipid, and ash contents in the fresh tendon of tuna tail and the extracted collagens were analyzed according to the AOAC methods [22].

#### 2.4.2. Collagen Extraction Yield

The yields of tuna-tail-tendon collagens were calculated using a following equation:

Yield (%) = [Dried collagen weight (g)/Tuna tail tendon used for extraction (g)] × 100(1)

#### 2.4.3. Color Determination

Colors of tuna-tail-tendon collagens with parameters consisting of L*, a*, b* were determined using a Hunter Lab Colorimeter (ColorFlex EZ Spectrophotometer, Reston, VA, USA). The color difference (∆*E*) was calculated using the following equation.

∆*E* = [(∆L*)2 + (∆a*)2 + (∆b*)2]^1/2^ (2)

where ∆L*, ∆a*, and ∆b* are the difference between the color parameters of samples and those white standard plate (91.40 of L*, −1.37 of a*, −0.33 of b*).

#### 2.4.4. Protein Solubility

The protein solubility of tuna-tail-tendon collagens was measured in 0.5 M acetic acid. In brief, the 200 mg collagen samples were dissolved in 10 mL of 0.5 M acetic acid with gentle stirring at 4 °C to obtain final concentrations of 20 mg/mL. The solution was then centrifuged at 9000× *g* at 4 °C for 30 min. The supernatant was determined the protein using Lowry et al. [23] method. The protein solubility was calculated in mg soluble protein/mg collagens. Bovine serum albumin (Sigma-Aldrich Co., St. Louis, MO, USA) was used as a standard protein. 

#### 2.4.5. The Effect of NaCl on Collagen Solubility

The effect of NaCl concentration on collagen solubility was evaluated using the modified method of Bae et al. [24]. The 5 mL of collagen solution (3 mg/mL) was mixed with 5 mL of NaCl in 0.5 M acetic acid at various concentrations (0–6 M). The mixtures were centrifuged at 9000× *g* for 30 min at 4 °C. Protein content in the supernatants was measured according to previous mention in Section 2.4.4. Relative solubility was calculated in comparison with that obtained at the NaCl giving the highest solubility using the following equation:

Solubility (%) = [Protein concentration in supernatant (mg/mL)/Highest protein concentration in supernatant (mg/mL)] × 100(3)

#### 2.4.6. The Effect of pH on Collagen Solubility

The effect of pH on tuna-tail-tendon-collagen solubility was determined using a modified method of Jongjareonrak et al. [6]. The extracted collagen was dissolved in 0.5 M acetic acid with 3 mg/mL and the pH was adjusted using either 6 M HCl or 6 M NaOH to obtain a final pH range of 1–10. The mixtures were centrifuged at 9000× *g* for 30 min at 4 °C. Protein content in the supernatants was measured according to the description in Section 2.4.4. Relative solubility was calculated in comparison with that obtained at the pH giving the highest solubility using the Equation (3).

#### 2.4.7. Fourier Transform Infrared Spectroscopy (FTIR)

The FTIR spectra were obtained using a Fourier Transform Infrared (FTIR) Spectrometer (NICOLET 6700FT-IR, Thermo Fisher Scientific, Waltham, MA, USA) following Huang et al.’s [13] method. Spectra were acquired at a resolution of 4000–400 cm^−1^ with a resolution of 4 cm^−1^.

#### 2.4.8. Scanning Electron Microscope (SEM)

The surface microstructure of lyophilized collagen from tuna-tail tendon was observed using SEM (JSM-IT300, JEOL Ltd., Tokyo, Japan) following the method of Zhang et al. [25]. Collagen sample was affixed in a standard SEM sample holder, coated with gold using an automatic fine coater (JFC1600; JEOL Ltd., Tokyo, Japan), and subjected to the specimen chamber; the distance between the camera and the workpiece is 12.5 mm, beam-spot size 30 was then used to obtain the surface microstructure.

#### 2.4.9. Viscosity of Collagen Solution

The viscosity of collagen solution at several temperatures and the denaturation temperature were determined according to a modified method of Zhang et al. [26]. The 3 mg of extracted collagens was dissolved in the 100 mL of 0.5 M acetic-acid solution. The viscosity was measured using viscometer (Brookfield, DV-2T, Middleboro, MA, USA) with spindle No. 2 and 100 rpm of speed. The solution was heated from 10 to 50 °C with 5 °C/min of heating rate. At the designated temperature, the solution was held for 10 min prior to viscosity determination. The measurement was done three times at each point. The fractional viscosity at the given temperature was calculated by the following equation:

Fractional viscosity = (V_(T)_ − V_(50°C)_)/(V_(10°C)_ − V_(50°C)_)

where V_(T)_ is viscosity at the given temperature, V_(50°C)_ is viscosity at 50 °C, and V_(10°C)_ is viscosity at 10 °C. The fractional-viscosity profile was plotted against the temperatures. The temperature at the point of 0.5 fractional viscosity was the denaturation temperature (T_d_).

#### 2.4.10. Differential Scanning Calorimetry (DSC)

The DSC was done using a differential-scanning-calorimeter model DSC3+ (Mettler-Toledo, LLC., Columbus, OH, USA). Dry-collagen samples (20–25 mg) were placed and sealed in 40 µL aluminum pans to prevent mass loss. The samples were subjected to heat with a setting on the DSC scan at 10 °C/min over the range of 0–200 °C under nitrogen atmosphere (50 mL N_2_/min). A 40 µL empty-aluminum pan was used as the reference. The onset temperature (T_onset_), the thermal denaturation peak temperature (T_max_), and the end-set temperature (T_endset_) were estimated from the thermograms.

#### 2.4.11. SDS-Polyacrylamide Gel Electrophoresis (SDS–PAGE)

SDS-PAGE analysis was done according to the method of Laemmli [27]. The collagen sample was dissolved in 5% SDS solution. The mixtures were dialyzed against deionized water overnight at 4 °C, followed by centrifugation at 6000 rpm for 15 min to remove undissolved debris. The sample buffer (0.5 M Tris–HCl, pH 6.8 containing 2% SDS and 50% glycerol in the presence of 0.02% (*v*/*v*) β-ME were mixed with the solubilized sample (1:1, *v*/*v*). Samples were loaded onto a polyacrylamide gel with 7.5% separating gel and 4.5% stacking gel and were then subjected to electrophoresis at a constant current of 20 mA/gel. After electrophoresis, the gels were stained with 0.1% (*w*/*v*) Coomassie blue R-250 in 30% (*v*/*v*) methanol and 7.5% (*v*/*v*) acetic acid for 30 min. Finally, the gels were de-stained with a mixture solution of 30% (*v*/*v*) methanol and 10% (*v*/*v*) acetic acid for 1 h. The high MW protein marker was used for estimation of protein MW. Type I collagen from calf skin (Sigma-Aldrich) was used as a standard.

#### 2.4.12. Amino Acid Determination

Amino acids were determined according to the method described by Jimenez-Martín et al. [28] with a slight modification. The 100 mg of collagens were hydrolyzed by 5 mL of the mixture-hydrolysis solution containing 6 N HCl, 5% thioglycolic acid, and 0.1% phenol. This hydrolysis was done at 110 °C for 18 h in a hot air oven. After hydrolysis, 1 mL of hydrolyzed sample was centrifuged at 10,000× *g* for 10 min. An aliquot (100 µL) of the supernatant was neutralized with 1 M sodium carbonate until pH was in the range of 1.0–2.0. The neutralized mixture (25 µL) was transferred to the 2 mL GC glass vial, which was subsequently added with 50 µL of norleucine (200 nmol/mL) as an internal standard. The mixture was dried at 60 °C about 1–2 h, added the 50 µL of dichloromethane, and re-dried for 30 min in order to remove the residual water. Dried samples were derivatized by adding 50 µL of derivatizing agent, including the MTBSTFA with 1% TBDMSCl (Sigma-Aldrich) and 50 µL of acetonitrile. Next, the sample vials were sealed with an aluminum cap with PTFE/Red Rubber Septa, then incubated at 100 °C for 4 h in a hot-air oven, and cooled at room temperature. The derivatized sample was analyzed using gas chromatography (Agilent 7890B, Agilent, Santa Clara, CA, USA) equipped with mass spectrometer (Agilent, 7000D) and PAL auto-sampler system (CTC Analytics AG, Zwingen, Switzerland). Aliquots of the derivatized amino acids (2 µL) were injected by using pulsed split mode at 1:5 split ratio at 280 °C into a HP-5MS column (30 m, 0.25 mm of Ø, Agilent J&W GC column). Helium was used as a carrier gas with a constant flow rate of 1.4 mL/min. The GC oven was programmed as follows: ramp from 130–190 °C (6 °C/min) and to 230 °C (30 °C/min), held at 230 °C for 5 min, then ramp to 325 °C, and held at 325 °C for 6 min. The transfer line, ion source (EI), and quadrupole were set as 325 °C, 240 °C, and 180 °C, respectively. The mass spectrometer was operated in selected ion monitoring (SIM) mode. The calibration curves were done using the mixture of all amino acids with different concentrations ranged from 25 to 400 nmol/mL. Samples were analyzed in triplicate. 

### 2.5. Antioxidant Activities of Tuna Tail Tendon Collagen

#### 2.5.1. DPPH Radical Scavenging Activity (DPPH)

The DPPH radical scavenging activity was determined using the method of Doungapai et al. [29] with a slight modification. The 100 µL of collagen solution was mixed with 100 µL 0.05 mmol/L DPPH solution in 95% ethanol, incubated in the dark for 30 min. The absorbance at 515 nm was obtained using Varioskan Lux microplate reader (Thermo Fisher Scientific Inc., Singapore). The distilled water was used for the control. The scavenging-activity plot was created using the tuna-tail-tendon collagen concentration versus scavenging activity. The IC_50_ or the concentration obtaining 50% DPPH radical scavenging activity was estimated from the plot using an equation from regression analysis. The DPPH radical scavenging activity was expressed following equation:

DPPH radical scavenging activity (%) = [(A_0_ − A_1_)/A_0_] × 100

when A_0_ is the obtained absorbance from DPPH solution and A_1_ is the obtained absorbance of collagen solution mixed with DPPH solution. 

#### 2.5.2. ABTS Radical Scavenging Activity (ABTS)

The ABTS radical scavenging activity was performed based on the method of Upata et al. [30]. The stock solution generated by mixing 7 mmol/L ABTS and 2.45 mM K_2_S_2_O_8_ and keeping it in the dark at 4 °C for 16–18 h. The working solution was prepared by dilution of the stock solution with 96% ethanol to obtain 0.70 ± 0.02 of absorbance at 734 nm. The sample solution (20 µL) was mixed with ABTS working solution (200 µL) and kept in the dark at room temperature for 8 min. The absorbance of the mixture was measured at 734 nm using a Varioskan Lux microplate reader (Thermo Fisher Scientific Inc.). ABTS scavenging activity and the IC_50_ values were calculated in a similar manner as used for the DPPH analysis. 

#### 2.5.3. FRAP Reducing Antioxidant Power (FRAP)

The FRAP assay was conducted following Wangtueai et al. [31]. The FRAP working solution was a mixture of 25 mL of 300 mM acetate buffer (pH 3.6), 2.5 mL of 10 mM FeCl_3_·6H_2_O, and 2.5 mL of 10 mM TPTZ in 40 mM of HCl. The solution was kept at 37 °C for 30 min. A 10 µL sample (10 mg/mL) was mixed with FRAP working solution (200 µL) and kept in the dark for 30 min. The absorbance was measured at 593 nm using a Varioskan Lux microplate reader. The solution of FeSO_4_·7H_2_O was used as a standard. Results were expressed in mmol FeSO_4_/g sample. Additional dilutions were done if the FRAP value was over the linear range of the standard curve.

#### 2.5.4. Hydroxyl Radical Scavenging Activity (OH)

The OH radical scavenging activity was executed following the method of Wang et al. [32]. The 2 mL of collagen solution (10 mg/mL) and the 1 mL of 1.865 mmol/L 1,10-phenanthroline were mixed and added to 1 mL of 1.865 mmol/L FeSO_4_·7H_2_O and 1 mL 0.03% H_2_O_2_ (*v*/*v*). The mixture was stirred using a vortex mixture and kept at 37 °C for 60 min in the dark. The 200 µL of the mixture was taken to obtaining the absorbance at 536 nm using a Varioskan Lux microplate reader. The negative control was performed using distilled water to replace the sample solution. The blank used distilled water to replace H_2_O_2_. The OH radical scavenging activity was calculated from following equation:

Hydroxyl radical scavenging activity (%) = [(A_s_ − A_n_)/(A_b_ − A_n_)] × 100

where A_s_ is absorbance of sample, A_n_ is absorbance of negative control and A_b_ is absorbance of blank after reaction.

### 2.6. Statistical Analysis

The experimental design was performed with a completely randomized design (CRD). All experiments and analyses were conducted in triplicates. Analysis of variance (ANOVA) was performed and Duncan’s new multiple range tests (DMRT) were used to test for the differences between means (*p* ≤ 0.05) using the Minitab Statistical Software (Trial version 18, Solution Center, Bangkok, Thailand).

## 3. Results

### 3.1. Proximate Composition of Tuna Tail Tendon

The proximate compositions of fresh tuna-tail tendon are shown in Table 1. According to the compositions, tail tendon of tuna was found to contain a fairly high content of moisture (71.08 ± 1.03%) and protein (21.67 ± 0.38). Previous report found that the most protein content in tuna-tail tendon has been found >82% dry weight of type I collagen [4]. In addition, the low-fat (4.22 ± 0.05%) and ash (0.18 ± 0.03%) contents were determined. Then tuna-tail tendon was a potential source to be a raw material for collagen extraction. 

### 3.2. Yield of Collagen from Tuna Tail Tendon

The yield of collagens extracted from the tuna-tail tendon are shown in Table 2. The pepsin-solubilized collagen (APTC) was the highest (*p* ≤ 0.05), while acid-solubilized collagens using acetic acid (ATC) and vinegar (VTC) extractions were not significantly different (*p* > 0.05). Collagen molecule contains non-helical sections or telopeptide regions in the two terminal ends and the inter-molecular cross-linked structure, then it possesses the limitation of the solubility in acid solution [33]. However, the APTC had the highest yield that might be due to the effect of pepsin-assisting acid to specifically cut in N- and C-terminal of tropocollagen or cleavage the telopeptide zone, resulting a good solubility in the presence of acid [21]. A previous study observed that the extracted yield of collagens from various raw materials, such as fish skin, fish bones, and fish scales, varied with the range of 2–50% [34]. 

### 3.3. Proximate Composition of Collagens from Tuna Tail Tendon 

The lyophilized collagens obtained from tuna-tail tendon were used to determine the proximate compositions, as shown in Table 2. The content of protein, moisture, lipid, and ash of those three collagens (VTC, ATC, and APTC) were not significantly different (*p* > 0.05). All tuna-tail-tendon collagens were high in protein and low in lipid and ash contents. Protein content of VTC, ATC, and APTC were not significantly different, at about 91, 90, and 90% (wet weight basis), respectively. The moisture, lipid, and ash contents were in the range of 7.4–7.7%, 1.2–1.5%, and 0.34–0.35% (wet weight basic), respectively. The extraction process obtained a collagen with a high-protein content and water was excluded from the freeze-drying process. These collagens were low in lipid content (<1.5%), which might be due to salt-solution washing in the pretreatment process, capable of fat removing. A solution having slight polarity was enhanced by removing of lipids in raw material containing membrane lipids, such as phospholipids [35]. 

### 3.4. Color

Color value of collagens are shown in Table 2. The L*, a*, b*, and ∆E* of three collagens were significantly different (*p* ≤ 0.05). The VTC had more white color with the highest L* value (~79). The yellowish color of ATC and APTC was more than the VTC. Generally, satisfactory collagen should have little and brighter color that is conducive to further application [17]. The color of freeze-dried collagen shows unique characteristics in appearance and relates to the intended applications; a light color is easier to incorporate into any compositions without imparting any strong color into the products [36]. However, these three tuna-tail-tendon collagens had low-fat content, which obtained less yellow (b*) or red (a*) color value. This coincides with a previous study of Nilsuwan et al. [37] which explained that the remaining lipid in resulting collagen might undergo oxidation via Maillard reaction, leading to discoloration of collagen. In addition, collagenous raw materials and extraction methods affect collagen color [36].

### 3.5. Protein Solubility

The protein solubility of ATC, VTC, and APTC are shown in Table 3. These were not significantly different in terms of protein solubility among the three different extraction methods (*p* > 0.05). However, APTC showed a slightly better solubility than ATC and VTC. This might be due to the pepsin-aid extraction that obtained a smaller MW collagen that is slightly better for protein solubility. A previous report showed that a lower MW of collagen extracted from threadfin-bream skin using pepsin from the stomach of skipjack tuna was obtained. This was due to pepsin being hydrolyzed in both regions of the telopeptide and within tropocollagen [38].

### 3.6. The Effect of NaCl on Protein Solubility

**Figure 1:**
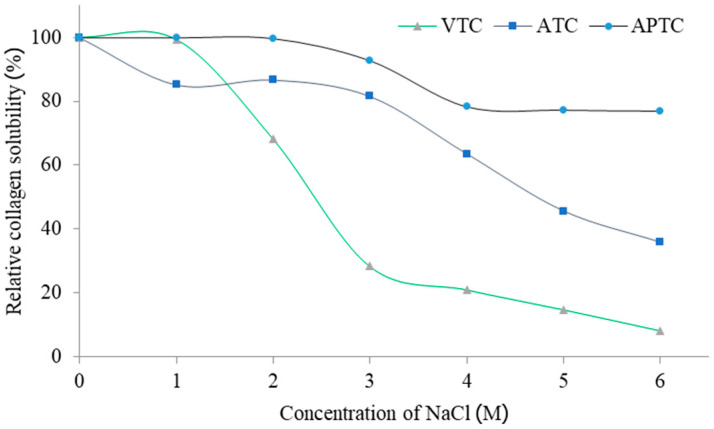
The effect of NaCl on protein solubility of tuna-tendon collagens (VTC: acid-solubilized collagen using vinegar extraction, ATC: acid-solubilized collagen using acetic-acid extraction, and APTC: pepsin-solubilized collagen).

The effect of NaCl content on protein solubility of collagen extracted from tuna-tail tendons is shown in Figure 1. The results were found that the increase in concentration of NaCl in the range of 1 to 6 mol/L decreased the protein solubility. This might be due to salt dissociation into a positive charge which binds with the protein (increase in ionic strength); then, the protein would be coagulated and precipitated, resulting in separation from the solution by the hydrophobic-hydrophobic interaction called salting out [39]. The acid -solubilized collagen extracted with vinegar (VTC) had the lowest solubility and pepsin solubilized collagen (APTC) had the highest solubility which may be influenced by the MW of the extracted collagen. It is also in accordance with MW, ATC, and APTC, which had a smaller MW than VTC; therefore, it had a higher solubility in high salt concentrations. The solubility behavior in different NaCl concentrations may play a major role in the extraction and application of collagens [7].

### 3.7. The Effect of pH on Protein Solubility

**Figure 2:**
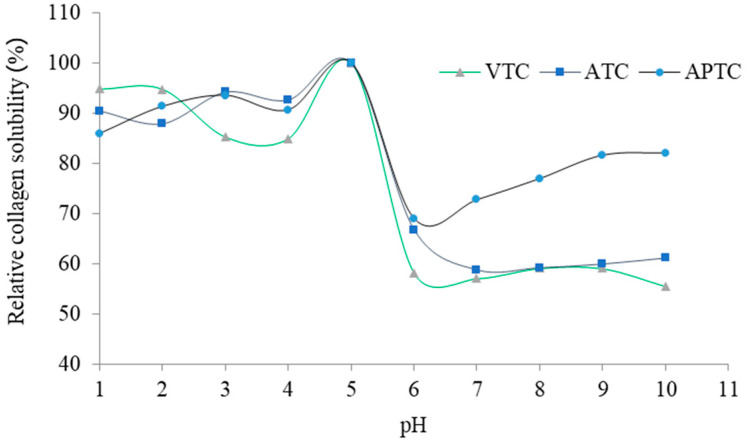
The effect of pH on protein solubility of tuna-tendon collagens (VTC: acid-solubilized collagen using vinegar extraction, ATC: acid-solubilized collagen using acetic-acid extraction, and APTC: pepsin-solubilized collagen).

The effect of pH content on protein solubility of collagen extracted from tuna-tail tendons is shown in Figure 2. All collagens were of good solubility with a pH range of 1–5 (*p* ≤ 0.05) and had lower solubility with a neutral pH. However, solubility slightly increased when the pH increased (Figure 2). This indicated that the pH of extracted collagen reached the pI value (isoelectric point), causing the protein to have a net positive and negative charge that created a repulsion force between the charges, resulting in protein precipitation [6]. A similar study is conducted by Chuaychan et al. [5], who examined the solubility of sea-bass-scale collagen using acid and pepsin. The solubility was high in the pH range of 1–4, while the pH range of 6–7 showed a significant reduction in solubility due to a relation of the reaction of hydrophobic-amino groups in collagen molecule [6]. This also corresponded to the solubility of collagen extracted from bighead-carp scales [7], brush-tooth lizardfish, and fish horse mackerel scales [40]. However, there was a small increase in solubility in the pH range of 8–10, which might be due to the extraction of collagen using acid and pepsin that may have a repulsive effect on the structure in the collagen molecule when the pH was higher than the pI [5,18,41]. Additionally, a difference in the solubility of collagen in different pH conditions might be a result of differences in MW and structures of collagen [41].

### 3.8. Fourier Transform Infrared Spectra (FTIR)

**Figure 3:**
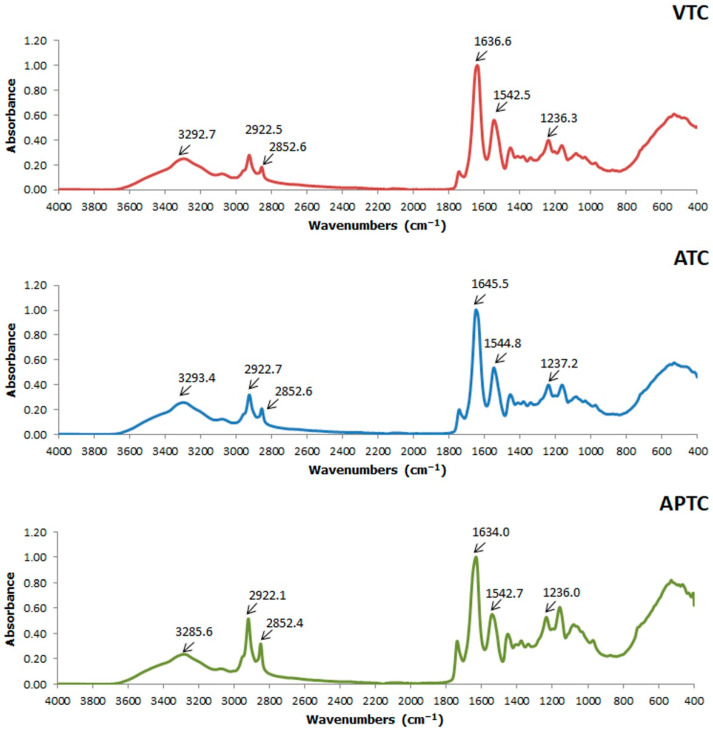
The FTIR spectra wavenumber of tuna-tail-tendon collagens (VTC: acid-solubilized collagen using vinegar extraction, ATC: acid-solubilized collagen using acetic-acid extraction, and APTC: pepsin-solubilized collagen).

The FTIR spectra of collagen extracted from tuna-tail tendons are shown in Figure 3. FTIR spectrums are connected to the amide groups, which lead to the auxiliary structure of polypeptide chains and the arrangement of acids within the collagen structure [42]. According to the report of Zhang et al. [25], they explained that the five major bands of FTIR spectra (amide A, amide B, amide Ι, amide ΙI, and amide ΙΙI band) represent the characteristic of amino acids and the high-proportion of proline and hydroxyproline acids of intact collagen molecules. In our findings, all five amide bands of three extracted collagens were within the specified range and indicated tuna-tail-tendon collagen as Type I collagen. The wavenumber of amide A, amide B, amide Ι, amide ΙI, and amide ΙΙI bands were in the range of 3286–3293 cm^−1^, 2853–2923 cm^−1^, 1634–1646cm^−1^, 1543–1545 cm^−1^, and 1236–1237cm^−1^, respectively. Generally, amide A bands are involved in hydrogen bonding at N-H position; if N-H position formed hydrogen bonds, the wavenumbers would be in the range of approximately 3300 cm^−1^; however, if N-H position did not form hydrogen bonds, the wavenumbers would appear in the range 3400–3440 cm^−1^. Amide B bands were found at wavenumbers 2700–3100 cm^−1^, showing asymmetrical stretch of the CH_2_ groups [43]. Amide I bands were found at wavenumbers 1600–1720 cm^−1^, which was related to the secondary structure of protein and the vibration of the carbonyl group elongation in the peptide. Amide II bands were found at wavenumbers 1480–1580 cm^−1^, indicating C-N group elongation and the N-H group flexion. Amide III bands were found at wavenumbers 1180–1300 cm^−1^, showing elongation of the C-N and N-H groups, as well as the triple-helix structure of the collagen [42].

### 3.9. Scanning Electron Microscope (SEM)

**Figure 4:**
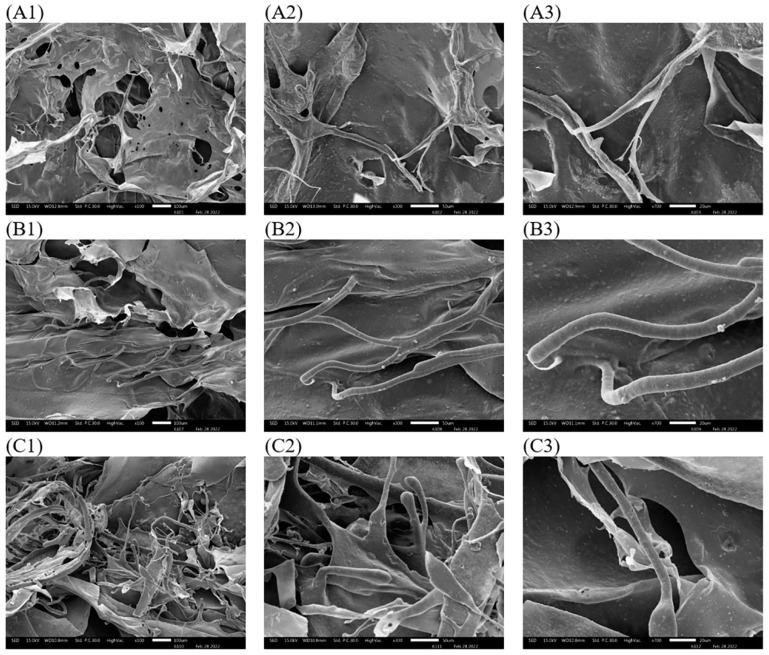
Surface microstructure of tuna-tendon collagens as VTC (acid-solubilized collagen using vinegar extraction) ×100 (**A1**), ×300 (**A2**), ×700 (**A3**); ATC (acid-solubilized collagen using acetic acid extraction) ×100 (**B1**), ×300 (**B2**), ×700 (**B3**); APTC (pepsin-solubilized collagen) ×100 (**C1**), ×300 (**C2**), ×700 (**C3**).

**Figure 6:**
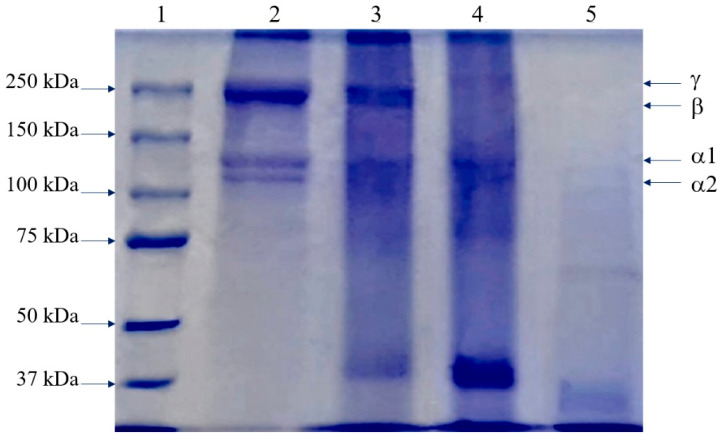
SDS–PAGE pattern of collagen from the tuna-tail tendon, Marker (1), Calf skin (2), VTC: acid-solubilized collagen using vinegar extraction (3), ATC: acid-solubilized collagen using acetic-acid extraction (4), APTC: pepsin-solubilized collagen (5).

The microstructure of collagen surfaces is important for the biomedical understanding of collagen [44]. For observation, the appearance of VTC, ATC, and APTC was identical to a soft white sponge with a porous and loose structure. This was similar to the collagens extracted from the skin of Amur sturgeon [45]. The micro-surface morphology of lyophilized tuna-tail-tendon collagens was observed using SEM as shown in Figure 4. The SEM images showed the surface collagen fibers with partial wrinkle, coarse, and uneven porous, probably due to freeze drying affected by dehydration process [45]. APTC was observed to have more small fibers than ATC and VTC; it might be due to a lower MW structure of APTC (Figure 6), resulting in acid and pepsin breakdown the triple helix structure of collagenous material. However, the overlapping structure of the collagen fibers are related to the wet-ability that are the ability to bind with water [21,25]. Moreover, the structural characteristics of the uniformly porous collagen particles were also beneficial for their application in biomedical products, such as the use of absorbent sponge-like properties and matric properties for cell formation [25,46].

### 3.10. Viscosity and Thermal Properties

**Figure 5:**
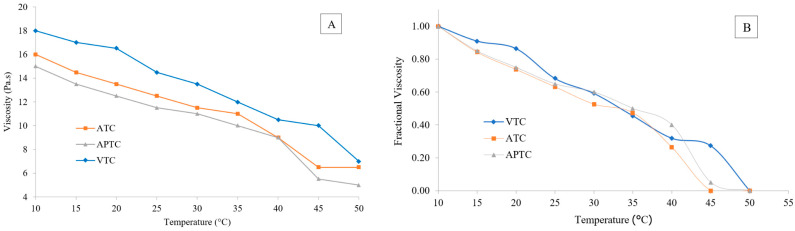
Viscosity (**A**) and fractional viscosity (**B**) of tuna-tail-tendon collagen (VTC: acid-solubilized collagen using vinegar extraction, ATC: acid-solubilized collagen using acetic-acid extraction, and APTC: pepsin-solubilized collagen).

Changes in viscosity of tuna-tail-tendon collagens upon heating are shown in Figure 5A. Among these three collagens, VTC had the highest viscosity (*p* ≤ 0.05) and ATC and APTC were not significantly different (*p* > 0.05). Viscosity drastically decreased with increasing temperature. VTC, ATC, and APTC showed similar changes in viscosity upon heating. Viscosity is one important parameter for collagen characteristic and normally relates to the MW of collagens, especially the proportion of β and γ chains; high MW leads to a high viscosity [47]. A previous study of Normah and Maidzatul Afiqah [48], which reported that sin-croaker collagen represented broader band of β and γ chains in a SDS-PAGE study, also showed that the collagen had a slightly higher viscosity, compared with collagen showing narrow bands. In addition, collagen viscosity was related to the aggregation of collagen molecules resulting in the inter- or intra-protein molecular interactions [8]. The decrease in viscosity during heat-treatment may be due to a de-structure or depolymerization process of collagen molecule upon heating, the triple-helix structure within the collagen molecule that consists of hydrogen bonds in the polymer has gradually broken and transformed into the random coil configuration, resulting in the changing of physical properties [49,50].

Thermal denaturation temperature (T_d_) was calculated from a curve of fractional viscosity profile as present in Figure 5B. The three tuna-tail-tendon collagens exhibited a similar loss of viscosity with heating from 10 °C to 50 °C at a concentration of 0.3% (*w*/*v*). From a thermal denaturation curve, the T_d_ of VTC, ATC, and APTC were 34.1, 35.0, and 36.4, respectively. The previous studies showed various Td of fish collagens, depending on raw materials, such as 35–39 °C for eel-fish [42], 35–36 °C for squid skin [51], 32–33 °C for Amur sturgeon skin [43], 31–33 °C for bigeye tuna skin [15], and 29–30 °C for balloon fish skin [52], while 37 °C mammalian collagen is obtained from porcine collagen [44]. Besides, the difference of Td may also correlate with the collagen- extraction methods, environmental and body temperatures of the fishes, as well as the imino acid (proline and hydroxyproline) compositions of the collagen. The collagen containing higher content of imino acid result in higher denaturation temperature [26,52]. As proline and hydroxyproline are a part of tropocollagen (triple-helix) affecting collagen stability, proline that reacts hydroxylation becomes hydroxyproline forming hydrogen bonds with neighboring molecules, giving collagen to the ability of heat resistance [26].

### 3.11. DSC Thermogram

DSC and viscosity measurements are commonly used to assess the thermal stability of collagens [52]. Thermal properties are used for the information about the effect of processing temperature on the collagen structure during the manufacture processes [53]. DSC thermograms of the lyophilized-tuna-tail-tendon collagens (7.4–7.7% moisture content) are shown in Table 4. The peak maximum temperature (T_max_) of VTC, ATC, and APTC were 170.8, 168.0, and 168.5 °C, respectively. T_max_ values of these three collagens were not significantly different (*p* > 0.05). The T_max_ values were associated with the denaturation of the triple helix of collagen structure, the dissociation of hydrogen bonds, and side-molecules rearrangement into uncoiled structures [54]. Generally, the denaturation temperature of collagen with full hydration or solution is about 40 °C, while a relatively low hydration collagen is about 150 °C [53,54]. Gauza-Włodarczyk et al. [54] have reported that the denaturation point of fish-skin collagen in the form of gel, solid, or lyophilized forms are in the range of 25–35 °C and at about 147 °C, respectively. A solid or lyophilized form with relatively low hydration is shows a higher denaturation point; it might be due to the collagen containing relatively low water and has a compact structure with the triple-helix region joining together [55]. Although the stability of collagen may be influenced mainly by intra-molecular hydrogen bonds and/or hydrogen bonds from the neighboring regular water structure, cross-linked fibers are further stabilized by hydrophobic bonds, and the concentration or strength of these bonds is greater in dry fibers than swollen fibers [56].

### 3.12. SDS–PAGE

The SDS-PAGE patterns of the collagens extracted from tuna-tail tendon are shown in Figure 6. The band patterns of ATC and VTC were almost similar, which encountered γ, β, α1, and α2 chains in accordance with Type I collagen from calf skin. Three collagens from tuna-tail tendon contained α1 and α2 chains, suggesting that they belonged to type I collagen. It was noted that the intensity of γ and β bands of ATC was slightly lower than VTC as can be observed from the SDS-PAGE. This might be an effect of acetic-acid concentration in the extraction solution, resulting in a slight degradation in the protein pattern. The α1 and α2 bands of ATC and VTC had a similar MW of ~130 kDa and ~120 kDa, respectively. The APTC was observed to have low intensity of α1 and α2 bands, and lacked γ and β bands. This was presumably due to the effect of pepsin and acetic acid in the degradation of native collagen by cleaving at the cross-link-containing telopeptide that may remove some parts of these regions, resulting in a decrease in MW of the obtained collagens [5]. 

### 3.13. Amino Acid Profile

Amino-acid composition of collagens from tuna-tail tendon were expressed per 1000 total residues, as shown in Table 5. The stability, physicochemical, and nutritional properties of collagen are commonly influenced by amino-acid composition [37]. Collagen extracted from tuna-tail tendon (VTC, ATC and APTC) contained similar predominant amino acids with raking from glycine (161–200/1000 residues), glutamic acid (108–117/1000 residues), proline (79–89/1000 residues), alanine (77–88/1000 residues), hydroxyproline (48–68/1000 residues), and aspartic acid (63–76/1000 residues). In general, glycine is spaced at every third residue of α-chain (Gly-X-Y) in collagen except for the telopeptide zones, in which the X and Y positions are probably proline and hydroxyproline, respectively [57]. In the structure, glycine plays a key role in the formation of inter-chain hydrogen bonds in α-chain [58]. As mentioned above, the content of imino acids (proline and hydroxyproline) is generally correlated with the thermal stability of collagen, high imino-acid content, causing collagen to deteriorate at elevated temperatures [26,52]. In this research study, the imino-acid content of VTC, ATC, and APTC were about 157.2, 145.4, and 127.3 residues/1000 residues. While a variety of imino- acid content in fish collagens have been reported, such as sailfish skin (213–221/1000 residues), bighead carp byproducts (156–175/1000 residues), salmon skin (191–193/1000 residues), and swim bladder of yellowfin tuna (128–169/1000 residues) [7,20,21,37]. However, many factors affected amino-acid compositions in collagen, such as the species of aquatic animals used for extraction, pre-treatment, and extraction processes.

### 3.14. Antioxidant Activity of Tuna Tendon Collagen

The antioxidant activity of tuna-tail tendon collagens was evaluated using different assays (DPPH, ABTS, and OH- radical scavenging activities and FRAP assay). Antioxidant activities of ATC, APTC, and VTC are shown in Table 6. Antioxidant activity was observed in tuna-tail-tendon collagens may be corelated with amino acid and peptide composition and MW, such as hydrophobic-amino-acid groups (Table 6), which may most likely be involved in antioxidant activities. DPPH-free radicals reacted with antioxidant compounds or radical species, causing electrons to exchange which would lead to color change from a purple solution to a colorless solution, resulting in reduced light absorption [57]. The IC_50_ values for DPPH-radical scavenging activities of VTC, ATC, and APTC were not significantly different (*p* > 0.05). The IC_50_ values of those three collagens were within the range of 0.12–0.29 mg/mL, which were lower than collagens from yellowfin tuna skin (0.56 mg/mL) [17]. Lower IC_50_ value indicates a high antioxidant capacity. This might be due to the collagens consists hydrophobic amino acids such as valine and leucine at *N*-terminal, and/or containing of proline, histidine, or tyrosine within the peptide chain, resulting in a good antioxidant capacity. Previous studies have explained that the small MW peptides isolated from protein hydrolysate commonly had a stronger antioxidant [2,3] as well as a peptide containing threonine, phenylalanine, leucine, valine, glutamic, and alanine in the sequence that expressed strong antioxidant activities [29].

ABTS free radicals are nitrogen-centered radicals that are blue or green color. When the antioxidants, which has the ability to trap oxygen or inhibit oxidation, were added, the solution would turn to colorless [59]. The IC_50_ values of ABTS’ radical scavenging activities of ATC, APTC, and VTC were not significantly different (*p* > 0.05). However, it obtained a higher IC_50_ of ABTS’ radical scavenging activities than DPPH’s radical scavenging activities. This might be influenced by a difference in peptide length, amino-acid sequence, amino-acid composition, amino-acid side chain, structure, and hydrophobicity [60]. In addition, Senphan and Benjakul [61] reported that ABTS’ radical scavenging activities are widely used to assay the antioxidant activities, which can be applied to both hydrophilic and lipophilic compounds. 

The FRAP assay was used to determine the dwindling in ferric ion (Fe^3+^)-ligand complex to the intensely blue-colored ferrous (Fe^2+^) complex by antioxidants under acidic condition. This method absorbs light at a wavelength of 593 nm; then, it can be used to determine the total reduction power of antioxidants by comparing to the FeSO_4_.7H_2_O, which is a standard substance. The high FRAP value in high antioxidants is due to the fact that the sample had the ability to reduce Fe^3+^ to Fe^2+^ [62]. The FRAP assays of ATC and VTC were not significantly different (*p* > 0.05), but these two collagens showed a higher FRAP value than APTC (*p* ≤ 0.05). The stronger reduction powder indicated that it could donate an electron to free radicals, resulting in a capacity to prevent or retard in the state of propagation [63].

Hydroxyl radical (OH-) is an active free radical that can damage an important biomolecule in the body, such as DNA and proteins, through continuous chain reactions. A study demonstrated its ability to inhibit OH-free radicals, the pink color of the solution would fade due to the added antioxidant that inhibits free radical. Furthermore, there were many factors affecting OH-radical-antioxidant activities, such as hydrophobic-amino acids [64,65]. The OH-radical scavenging activities of ATC, APTC, and VTC were in the range of 94–97% inhibition and were not significantly different (*p* > 0.05) among three extracted collagens (Table 6).

## 4. Conclusions

Acid- and pepsin-solubilized collagen cloud was extracted from tuna tail-tendon by using acetic acid, commercial vinegar, and acetic acid, aided by pepsin. Tuna-tail tendon was identified as type I collagen. All extraction methods had a triple-helix structure and showed slight variations of thermal stability. ATC showed the highest yield and good solubility in NaCl. Additionally, collagens showed high solubility with pH 1–5 when applied in acidic foods. Moreover, tuna-tail-tendon collagen had antioxidant activities, whose clouds can be used as a collagen hydrolysate in order to increase the antioxidant activity for further application in dietary supplements or bioactive ingredients.

## Figures and Tables

**Table 1:** The proximate compositions of the tail tendon of tuna (*Katsuwonus pelamis*).

Sample	Proximate Compositions (% of Wet Weight)			
	Moisture	Protein	Fat	Ash
Tuna tail tendon	71.08 ± 1.03	21.67 ± 0.38	4.22 ± 0.05	0.18 ± 0.03

Note: Values are reported as mean ± SD from triplicate determinations.

**Table 2:** The yield, proximate compositions and color of tuna-tail tendon collagen from different three extraction methods.

Parameters	Tuna-Tail-Tendon Collagen		
	VTC	ATC	APTC
Yield (% wet weight basis)	7.88 ± 0.41 ^b^	8.67 ± 0.35 ^b^	12.0 ± 0.07 ^a^
Protein (% wet weight basis)	90.2 ± 0.29 ^a^	90.5 ± 0.07 ^a^	90.0 ± 0.74 ^a^
Moisture (% wet weight basis)	7.62 ± 0.59 ^a^	7.40 ± 0.06 ^a^	7.72 ± 0.73 ^a^
Lipid (% wet weight basis)	1.34 ± 0.16 ^a^	1.46 ± 0.16 ^a^	1.21 ± 0.27 ^a^
Ash (% wet weight basis)	0.35 ± 0.08 ^a^	0.34 ± 0.04 ^a^	0.35 ± 0.03 ^a^
Color			
L*	79.0 ± 0.18 ^a^	73.1 ± 0.05 ^c^	74.4 ± 0.20 ^b^
a*	4.89 ± 0.04 ^c^	6.35 ± 0.02 ^b^	8.28 ± 0.04 ^a^
b*	30.2 ± 0.25 ^c^	34.9 ± 0.10 ^a^	32.5 ± 0.13 ^b^
∆E*	34.3 ± 0.17 ^c^	41.3 ± 0.08 ^a^	39.1 ± 0.20 ^b^
Viscosity * (Pa.s)	14.3 ± 0.29 ^a^	12.5 ± 0.50 ^b^	11.5 ± 0.50 ^b^

Note: Values are reported as mean ± SD (n = 3). Different superscripts in the same row indicate the significant difference (*p* ≤ 0.05). * Viscosity of collagen solution in 0.5 M acetic acid at 25 °C. VTC: acid-solubilized collagen using vinegar extraction, ATC: acid-solubilized collagen using acetic acid extraction, and APTC: pepsin-solubilized collagen.

**Table 3:** The soluble-protein content of tuna-tail-tendon collagens.

Tuna Tail Tendon Collagen	Protein Solubility (mg/mg Collagen)
ATC	0.52 ± 0.07 ^a^
VTC	0.44 ± 0.03 ^a^
APTC	0.67 ± 0.12 ^a^

Note: Values are reported as mean ± SD (n = 3). VTC: acid-solubilized collagen using vinegar extraction, ATC: acid-solubilized collagen using acetic-acid extraction, and APTC: pepsin-solubilized collagen. Different superscripts in the same column indicate significant differences (*p* ≤ 0.05).

**Table 4:** DSC thermogram of tuna-tail-tendon collagens.

Tuna Tail Tendon Collagen	T_onset_	T_max_	T_endset_
VTC	155.2 ± 0.8 ^a^	170.8 ± 1.4 ^a^	180.4 ± 3.4 ^a^
ATC	150.3 ± 6.3 ^a^	168.0 ± 3.5 ^a^	176.9 ± 4.4 ^a^
APTC	154.7 ± 4.1 ^a^	168.5 ± 5.9 ^a^	178.6 ± 4.4 ^a^

Note: Values are reported as mean ± SD (n = 3). VTC: acid-solubilized collagen using vinegar extraction, ATC: acid-solubilized collagen using acetic-acid extraction, and APTC: pepsin-solubilized collagen. Similar superscripts in the same column indicate the non-significant differences (*p* > 0.05).

**Table 5:** Amino-acid composition of tuna-tail tendon collagens.

Amino Acids	Content (Residues/1000 Residues)		
	VTC	ATC	APTC
Alanine	87.11 ± 1.89	87.51 ± 3.28	77.22 ± 0.33
Glycine	199.6 ± 4.65	193.5 ± 13.9	161.4 ± 0.22
Valine	25.02 ± 1.08	25.99 ± 2.43	31.81 ± 0.76
Leucine	41.56 ± 1.19	44.48 ± 0.49	49.93 ± 2.18
Isoleucine	21.59 ± 0.48	24.45 ± 0.45	33.43 ± 0.69
Proline	89.44 ± 1.41	81.85 ± 0.78	79.01 ± 2.60
Methionine	25.91 ± 0.16	25.29 ± 1.17	28.18 ± 1.85
Serine	29.08 ± 4.37	31.94 ± 1.42	34.12 ± 2.42
Threonine	35.95 ± 0.13	37.51 ± 2.24	43.13 ± 0.81
Phenylalanine	23.61 ± 2.14	27.08 ± 0.38	28.08 ± 0.61
Aspartic acid	62.77 ± 1.58	63.57 ± 0.34	75.47 ± 1.00
Hydroxyproline	67.86 ± 3.81	63.74 ± 3.36	48.32 ± 5.12
Cysteine	10.05 ± 0.41	11.13 ± 0.27	9.31 ± 0.58
Glutamic acid	115.1 ± 1.64	107.6 ± 1.65	117.0 ± 1.20
Ornithine/Arginine	25.30 ± 0.37	20.79 ± 3.28	24.11 ± 0.21
Lysine	44.34 ± 0.28	43.22 ± 3.94	49.59 ± 0.50
Histidine	17.26 ± 1.23	20.05 ± 0.19	21.88 ± 0.73
Tyrosine	12.38 ± 1.30	16.73 ± 1.14	23.03 ± 1.87
Tryptophan	1.02 ± 0.09	3.33 ± 1.15	6.16 ± 0.84
Cystine	65.07 ± 1.04	70.23 ± 3.28	58.81 ± 1.82
Total amino acids	1000	1000	1000
Imino acid	157.2	145.4	127.3

Note: Values are reported as mean ± SD (n = 3). VTC is acid-solubilized collagen using vinegar extraction, ATC is acid-solubilized collagen using acetic-acid extraction, and APTC is pepsin-solubilized collagen.

**Table 6:** The antioxidant activity of tuna-tail-tendon collagens.

Collagen	Antioxidant Activities			
	DPPH IC_50_ (mg/mL)	ABTS IC_50_ (mg/mL)	FRAP (mmol FeSO_4_/mL)	OH (% Inhibition at Concentration 10 mg/mL)
VTC	0.286 ± 0.119 ^a^	19.2 ± 1.07 ^ab^	0.118 ± 0.009 ^a^	96.7 ± 0.11 ^a^
ATC	0.124 ± 0.023 ^a^	14.6 ± 0.95 ^b^	0.118 ± 0.004 ^a^	94.2 ± 3.01 ^a^
APTC	0.293 ± 0.091 ^a^	17.7 ± 2.39 ^ab^	0.085 ± 0.001 ^b^	96.8 ± 2.66 ^a^

Note: Values are reported as mean ± SD (n = 3). Different superscripts in the same column indicate the significant differences (*p* ≤ 0.05). VTC is acid-solubilized collagen using vinegar extraction, ATC is acid-solubilized collagen using acetic-acid extraction, and APTC is pepsin-solubilized collagen.

## Data Availability

Data are reported in the article.

## References

[1] Mata W., Chanmalee T., Punyasuk N., Thitamadee S. (2020). Simple PCR-RFLP detection method for genus- and species-authentication of four types of tuna used in canned tuna industry. Food Control.

[2] Mongkonkamthorn N., Malila Y., Yarnpakdee S., Makkhun S., Regenstein J.M., Wangtueai S. (2020). Production of protein hydrolysate containing antioxidant and angiotensin -I-converting enzyme (ACE) inhibitory activities from tuna (*Katsuwonus pelamis*) blood. Processes.

[3] Mongkonkamthorn N., Malila Y., Regenstein J.M., Wangtueai S. (2021). Enzymatic hydrolysis optimization for preparation of tuna dark meat hydrolysate with antioxidant and angiotensin I-converting enzyme (ACE) inhibitory activities. J. Aquat. Food Prod. Technol..

[4] Shadwick R.E., Rapoport H.S., Fenger J.M. (2002). Structure and function of tuna tail tendons. Comp. Biochem. Physiol. Part A.

[5] Chuaychan S., Benjakul S., Kishimura H. (2015). Characteristics of acid- and pepsin-soluble collagens from scale of seabass (*Lates calcarifer*). LWT-Food Sci. Technol..

[6] Jongjareonrak A., Benjakul S., Visessanguan W., Nagai T., Tanaka M. (2005). Isolation and characterisation of acid and pepsin-solubilised collagens from the skin of Brownstripe red snapper (*Lutjanus vitta*). Food Chem..

[7] Liu D., Liang L., Regenstein J.M., Zhou P. (2012). Extraction and characterization of pepsin-solubilised collagen from fins, scales, skins, bones and swim bladders of bighead carp (*Hypophthalmichthys nobilis*). Food Chem..

[8] Tang C., Zhou K., Zhu Y., Zhang W., Xie Y., Wang Z., Zhou H., Yang T., Zhang Q., Xu B. (2022). Collagen and its derivatives: From structure and properties to their applications in food industry. Food Hydrocoll..

[9] Kozlowska J., Sionkowska A., Skopinska-Wisniewska J., Piechowicz K. (2015). Northern pike (*Esox lucius*) collagen: Extraction, characterization and potential application. Int. J. Biol. Macromol..

[10] Felician F.F., Xia C., Qi W., Xu H. (2018). Collagen from marine biological sources and medical applications. Chem. Biodivers..

[11] Liu Y., Ma D., Wang Y., Qin W. (2018). A comparative study of the properties and self-aggregation behavior of collagens from the scales and skin of grass carp (*Ctenopharyngodon idella*). Int. J. Biol. Macromol..

[12] Avila Rodriguez M.I., Rodriguez Barroso L.G., Sánchez M.L. (2018). Collagen: A review on its sources and potential cosmetic applications. J. Cosmet. Dermatol..

[13] Huang C.Y., Kuo J.M., Wu S.J., Tsai H.T. (2016). Isolation and characterization of fish scale collagen from tilapia (*Oreochromis* sp.) by a novel extrusion–hydro-extraction process. Food Chem..

[14] Regenstein J., Zhou P. (2007). Collagen and gelatin from marine by-products. Maximising the Value of Marine by-Products.

[15] Ahmed R., Haq M., Chun B.S. (2019). Characterization of marine derived collagen extracted from the by-products of bigeye tuna (*Thunnus obesus*). Int. J. Biol. Macromol..

[16] Lin X., Chen Y., Jin H., Zhao Q., Liu C., Li R., Yu F., Chen Y., Huang F., Yang Z. (2019). Collagen extracted from bigeye tuna (*Thunnus obesus*) skin by isoelectric precipitation: Physicochemical properties, proliferation, and migration activities. Mar. Drugs.

[17] Nurilmala M., Hizbullah H.H., Karnia E., Kusumaningtyas E., Ochiai Y. (2020). Characterization and antioxidant activity of collagen, gelatin, and the derived peptides from yellowfin tuna (*Thunnus albacares*) skin. Mar. Drugs.

[18] Yu D., Chi C.F., Wang B., Ding G.F., Li Z.R. (2014). Characterization of acid-and pepsin-soluble collagens from spines and skulls of skipjack tuna (*Katsuwonus pelamis*). Chin. J. Nat. Med..

[19] Ding D.D., Du B., Zhang C., Zaman F., Huang Y. (2019). Isolation and identification of an antioxidant collagen peptide from skipjack tuna (*Katsuwonus pelamis*) bone. RSC Adv..

[20] Kaewdang O., Benjakul S., Kaewmanee T., Kishimura H. (2014). Characteristics of collagens from the swim bladders of yellowfin tuna (*Thunnus albacares*). Food Chem..

[21] Tamilmozhi S., Veeruraj A., Arumugam M. (2013). Isolation and characterization of acid and pepsin-solubilized collagen from the skin of sailfish (*Istiophorus platypterus*). Food Res. Int..

[22] AOAC (2000). Official Methods of Analysis.

[23] Lowry O.H., Rosebrough N.J., Farr A.L., Randall R.J. (1951). Protein measurement with folin phenol reagent. J. Biol. Chem..

[24] Bae I., Osatomi K., Yoshida A., Osako K., Yamaguchi A., Hara K. (2008). Biochemical properties of acid-solubilized collagens extracted from the skins of underutilised fishes. Food Chem..

[25] Zhang Q., Wang Q., Lv S., Lu J., Jiang S., Regenstein J.M., Lin L. (2016). Comparison of collagen and gelatin extracted from the skins of Nile tilapia (*Oreochromis niloticus*) and channel catfish (*Ictalurus punctatus*). Food Biosci..

[26] Zhang Y., Liu W.T., Li G.Y., Shi B., Miao Y.Q., Wu X.H. (2007). Isolation and partial characterization of pepsin-soluble collagen from the skin of grass carp (*Ctenopharyngodon idella*). Food Chem..

[27] Laemmli U.K. (1970). Cleavage of structural proteins during the assembly of the head of bacteriophage T4. Nature.

[28] Jimenez-Martin E., Ruiz J., Perez-Palacios T., Silva A., Antequera T. (2012). Gas Chromatography-mass spectrometry method for the determination of free amino acids as their dimethyl-tert-butylsilyl (TBDMS) derivatives in animal source food. J. Agric. Food Chem..

[29] Doungapai C., Siriwoharn T., Malila Y., Autsavapromporn N., Makkhun S., Yarnpakdee S., Jantanasakulwong K., Regenstein J.M., Wangtueai S. (2022). UV-B Protective and antioxidant activities of protein hydrolysate from sea cucumber (*Holothuria scabra*) using enzymatic hydrolysis. Front. Mar. Sci..

[30] Upata M., Siriwoharn T., Makkhun S., Yarnpakdee S., Regenstein J.M., Wangtueai S. (2022). Tyrosinase inhibitory and antioxidant activity of enzymatic protein hydrolysate from jellyfish (*Lobonema smithii*). Foods.

[31] Wangtueai S., Siebenhandl-Ehn S., Haltrich D. (2016). Optimization of the preparation of gelatin hydrolysates with antioxidative activity from lizardfish (*Saurida* spp.) scales gelatin. Chiang Mai J. Sci..

[32] Wang B., Wang Y.M., Chi C.F., Luo H.Y., Deng S.G., Ma J.Y. (2013). Isolation and characterization of collagen and antioxidant collagen peptides from scales of croceine croaker (*Pseudosciaena crocea*). Mar. Drugs.

[33] Ahmed R., Getachew A.T., Cho Y.J., Chun B.S. (2018). Application of bacterial collagenolytic proteases for the extraction of type I collagen from the skin of bigeye tuna (*Thunnus obesus*). LWT-Food Sci. Technol..

[34] Jafari H., Lista A., Siekapen M.M., Ghaffari-Bohlouli P., Nie L., Alimoradi H., Shavandi A. (2020). Fish collagen: Extraction, characterization, and applications for biomaterials engineering. Polymers.

[35] Sae-leaw T., Benjakul S., O’Brien N.M. (2016). Effect of pretreatments and defatting of seabass skins on properties and fishy odor of gelatin. J. Food Biochem..

[36] Kumar P.G., Nidheesh T., Suresh P.V. (2015). Comparative study on characteristics and in vitro fibril formation ability of acid and pepsin soluble collagen from the skin of catla (*Catla catla*) and rohu (*Labeo rohita*). Food Res. Int..

[37] Nilsuwan K., Fusang K., Pripatnanont P., Benjakul S. (2022). Properties and characteristics of acid-soluble collagen from salmon skin defatted with the aid of ultrasonication. Fishes.

[38] Nalinanon S., Benjakul S., Visessanguan W., Kishimura H. (2008). Tuna pepsin: Characteristics and its use for collagen extraction from the skin of threadfin bream (*Nemipterus* spp.). J. Food Sci..

[39] Tan Y., Chang S.K.C. (2018). Isolation and characterization of collagen extracted from channel catfish (*Ictalurus punctatus*) skin. Food Chem..

[40] Minh-Thuy L.T., Okazaki E., Osako K. (2014). Isolation and characterization of acid-soluble collagen from the scales of marine fishes from Japan and Vietnam. Food Chem..

[41] Kittiphattanabawon P., Benjakul S., Visessanguan W., Nagai T., Tanaka M. (2005). Characterisation of acid-soluble collagen from skin and bone of bigeye snapper (*Priacanthus tayenus*). Food Chem..

[42] Oliveira V.D.M., Assis C.R.D., Costa B.D.A.M., Neri R.C.D.A., Monte F.T.D., Freitas H.M.S.D.C.V., França R.C.P., Santos J.F., Bezerra R.D.S., Porto A.L.F. (2021). Physical, biochemical, densitometric and spectroscopic techniques for characterization collagen from alternative sources: A review based on the sustainable valorization of aquatic byproducts. J. Mol. Struct..

[43] Yousefi M., Ariffin F., Huda N. (2017). An alternative source of type I collagen based on by-product with higher thermal stability. Food Hydrocoll..

[44] Veeruraj A., Arumugam M., Balasubramanian T. (2013). Isolation and characterization of thermostable collagen from the marine eel-fish (*Evenchelys macrura*). Process Biochem..

[45] Wang L., Liang Q., Chen T., Wang Z., Xu J., Ma H. (2014). Characterization of collagen from the skin of Amur sturgeon (*Acipenser schrenckii*). Food Hydrocoll..

[46] Mitra T., Sailakshmi G., Gnanamani A., Mandal A.B. (2012). Preparation and characterization of malonic acid cross-linked chitosan and collagen 3D scaffolds: An approach on noncovalent interactions. J. Mater. Sci. Mater. Med..

[47] Zhong R.L., Bin W., Chang F.C., Qi H.Z., Yan D.G., Jia J.T., Hong Y.L., Guo F.D. (2013). Isolation and characterization of acid soluble collagens and pepsin soluble collagens from the skin and bone of Spanish mackerel (*Scomberomorous niphonius*). Food Hydrocoll..

[48] Normah I., Maidzatul Afiqah M. (2018). Effect of extraction time on the physico-chemical characteristics of collagen from sin croaker (*Johniecop sina*) waste. Int. Food Res. J..

[49] Ahmad M., Benjakul S. (2010). Extraction and characterisation of pepsinsolubilised collagen from the skin of unicorn leatherjacket (*Aluterus monocerous*). Food Chem..

[50] Shaik M.I., Asrul Effendi N.F., Sarbon N.M. (2021). Functional properties of sharpnose stingray (*Dasyatis zugei*) skin collagen by ultrasonication extraction as influenced by organic and inorganic acid. Biocatal. Agric. Biotechnol..

[51] Veeruraj A., Arumugam M., Ajithkumar T., Balasubramanian T. (2015). Isolation and characterization of collagen from the outer skin of squid (*Doryteuthis singhalensis*). Food Hydrocoll..

[52] Huang Y.R., Shiau C.Y., Chen H.H., Huang B.C. (2011). Isolation and characterization of acid and pepsin solubilized collagens from the skin of ballon fish (*Diodon holocanthus*). Food Hydrocoll..

[53] Andonegi M., Correia D.M., Costa C.M., Lanceros-Mendez S., de la Caba K., Guerrero P. (2022). Tailoring physicochemical properties of collagen-based composites with ionic liquids and wool for advanced applications. Polymers.

[54] Gauza-Włodarczyk M., Kubisz L., Mielcarek S., Włodarczyk D. (2017). Comparison of thermal properties of fish collagen and bovine collagen in temperature range 298–670 K. Mater. Sci. Eng. C.

[55] Miles C.A., Ghelashvili M. (1999). Polymer-in-a-box mechanism for the thermal stabilization of collagen molecules in fibers. Biophys. J..

[56] Rochdi A., Foucat L., Renou J.P. (1999). Effect of Thermal denaturation on water–collagen interactions: NMR relaxation and differential scanning calorimetry analysis. Biopolymers.

[57] Benjakul S., Nalinanon S., Shahidi F., Simpson B.K. (2012). Fish Collagen. Food Biochemistry and Food Processing.

[58] Song Z., Liu H., Chen L., Chen L., Zhou C., Hong P., Deng C. (2021). Characterization and comparison of collagen extracted from the skin of the Nile tilapia by fermentation and chemical pretreatment. Food Chem..

[59] Rajnarayana K., Ajitha M., Gopireddy G., Giriprasad V.S. (2011). Comparative antioxidant potential of some fruits and vegetables using DPPH method. Int. J. Pharm. Technol..

[60] Intarasirisawat R., Benjakul S., Visessanguan W., Wu J. (2012). Antioxidative and functional properties of protein hydrolysate from defatted skipjack (*Katsuwonus pelamis*) roe. Food Chem..

[61] Senphan T., Benjakul S. (2014). Antioxidative activities of hydrolysates from seabass skin prepared using protease from hepatopancreas of Pacific white shrimp. J. Funct. Foods.

[62] Benzie I.F., Strain J.J. (1996). The ferric reducing ability of plasma (FRAP) as a measuring of antioxidant power: The FRAP assay. Anal. Biochem..

[63] Klomklao S., Benjakul S. (2018). Protein hydrolysates prepared from viscera of skipjack tuna (*Katsuwonus pelmanis*): Antioxidative activity and functional properties. Turk. J. Fish. Aquat. Sci..

[64] Rajapakse N., Mendis E., Jung W.K., Je J.Y., Kim S.K. (2005). Purification of a radical scavenging peptide from fermented mussel sauce and its antioxidant properties. Food Res. Int..

[65] Mathew S., Abraham T.E. (2006). In vitro antioxidant activity and scavenging effects of *Cinnamomum verum* leaf extract assayed by different methodologies. Food Chem. Toxicol..

